# Mechanical Performance of Fly Ash Based Geopolymer (FAG) as Road Base Stabilizer

**DOI:** 10.3390/ma15207242

**Published:** 2022-10-17

**Authors:** Liyana Ahmad Sofri, Mohd Mustafa Al Bakri Abdullah, Andrei Victor Sandu, Thanongsak Imjai, Petrica Vizureanu, Mohd Rosli Mohd Hasan, Mohammad Almadani, Ikmal Hakem Ab Aziz, Farahiyah Abdul Rahman

**Affiliations:** 1 Faculty of Civil Engineering & Technology, Universiti Malaysia Perlis (UniMAP), Arau 02600, Malaysia; 2 Centre of Excellence Geopolymer and Green Technology, (CEGeoGTech), Universiti Malaysia Perlis (UniMAP), Arau 01000, Malaysia; 3 Faculty of Chemical Engineering & Technology, Universiti Malaysia Perlis (UniMAP), Arau 01000, Malaysia; 4 Faculty of Material Science and Engineering, Gheorghe Asachi Technical University of Iasi, 41 D. Mangeron St., 700050 Iasi, Romania; 5 Romanian Inventors Forum, Str. Sf. P. Movila 3, 700089 Iasi, Romania; 6 National Institute for Research and Development for Environmental Protection INCDPM, 294 Splaiul Independentei, 060031 Bucharest, Romania; 7 Center of Excellence in Sustainable Disaster Management, School of Engineering and Technology, Walailak University, Nakhonsithammarat 80161, Thailand; 8 Technical Sciences Academy of Romania, Dacia Blvd. 26, 030167 Bucharest, Romania; 9 School of Civil Engineering, Engineering Campus, Universiti Sains Malaysia, Penang 14300, Malaysia; 10 Department of Civil Engineering, Faculty of Engineering—Rabigh Branch, King Abdulaziz University, Jeddah 21589, Saudi Arabia

**Keywords:** geopolymer, fly ash, base course material, road base stabilization, unconfined compressive strength

## Abstract

This study examines the strength development of fly ash-based geopolymer (FAG) as a stabilizer for road base material for pavement construction. In the last decade, there has been a rapid development of conventionally treated bases, such as cement-treated bases. However, a major problem with this kind of application is the shrinkage cracking in cement-treated bases that may result in the reflection cracks on the asphalt pavement surface. This study explores the effects of FAG on base layer properties using mechanistic laboratory evaluation and its practicability in pavement base layers. The investigated properties are flexural strength (FS), unconfined compressive strength (UCS), shrinkage, and resilient modulus (RM), as well as indirect tensile strength (ITS). The findings showed that the mechanical properties of the mixture enhanced when FAG was added to 80–85% of crushed aggregate, with the UCS being shown to be a crucial quality parameter. The effectiveness of FAG base material can have an impact on the flexible pavements’ overall performance since the base course stiffness directly depends on the base material properties. As a stabilizing agent for flexible pavement applications, the FAG-stabilized base appeared promising, predicated on test outcomes.

## 1. Introduction

The world has seen the tremendous expansion of road networks in emerging countries during the last several years. However, this rapid expansion has a serious implication since constructing roads consumes many crushed or virgin aggregates, especially for the development of base layers for roads [1]. A cement-treated base is an example of a stabilized base layer [2]. It consists of compacted mixtures of granular materials, Portland cement, and water. In current years, applying a cement-stabilized base has led to pavement damage, induced by the cement hydration process, as well as moisture loss [3]. Every year, cement output increases globally, and there are no signs that it will ever decline [4]. Since 1950, global cement production has multiplied 30 times, and nearly four-fold since 1990, making it the third largest source of anthropogenic carbon dioxide (CO_2_) emissions, after fossil fuels and land-use changes [5]. The production of cement requires enormous amounts of natural resources, for example, limestone, natural gas, fossil fuels, and electricity. High temperatures are necessary to manufacture cement in power plants, which results in 8 to 10 percent of carbon dioxide (CO_2_) being emitted into the environment [6]. The usage of cement may be reduced by utilizing several pozzolanic raw materials, including palm oil, rice husk ash, fly ash (FA) [7], fuel ash, bagasse ash [8], and phosphogypsum [9].

Geopolymer is a well-known environmentally friendly substance. It has been shown to have good characteristics. A geopolymer precursor material is composed of silicon (Si) and aluminum (Al) [10]. After a few steps, it would react with extremely alkaline activator solutions to produce a geopolymer binder [11]. With an alkaline activator solution, materials including blast furnace slag, metakaolin, and FA may be activated, leading to the development of geopolymerization [12]. Raw pozzolanic materials, for instance, FA, are widely used in geopolymer cement and concrete as aluminosilicate raw materials [13,14,15,16,17,18]. It can be applied as a replacement for or in addition to regular Portland cement in a variety of ways. FA application in cement provides benefits from an environmental and economic point of view since it possesses a zero-cost industrial waste that must be disposed of [19].

The most reliable primary raw material for developing alkali-activated materials is FA, whose activation has been the subject of extensive study over the past few decades [20]. It would positively impact the reduction of cement production and the development of sustainable building materials, as FA is a hazardous waste of electrical steam generated by burning coal [21]. Other widely used raw materials are kaolin, metakaolin, and bottom ash [22]. Previous studies have indicated that FA can also be utilized as a soil stabilizer for the sub-base layer in constructing roads [23]. FA is also excellent material for producing geopolymer concrete as a replacement for cement, and is suitable to be used as a road base stabilizer.

Studies have been carried out continuously to develop FA-based geopolymer (FAG) as an alternative binder. The strength, chemistry, morphological, and adhesion properties of FAG used as an asphalt binder modifier were investigated by Atmaja et al. [24]. Class F of FA was mixed with alkaline solutions (i.e., sodium hydroxide (NaOH) and sodium silicate (Na_2_SiO_3_)) to prepare the geopolymer. The modified bitumen’s impact values were higher than the base bitumen after adding the geopolymer. Overall, it produced the best results, and the ideal concentration for bitumen modification was 5% geopolymer. Dayal & Soundarapandian [25] investigated the properties of geopolymer-coated aggregates made of FA and their impact on the properties of bituminous mixes. The addition of a geopolymer paste based FA to a natural aggregate improved the physical and mechanical qualities of marginal aggregates, allowing them to fulfill the pavement construction requirements. According to the findings, geopolymers made with high calcium (Ca) content FA resulted in significantly enhanced aggregate strength properties in bituminous concrete mixtures produced with geopolymer-coated aggregates formed with FA.

In a separate study by Hu et al. [26], class F fly ash and red mud were combined with a sodium hydroxide and sodium silicate solution to generate a geopolymer stabilized base. According to the findings of the tests, both geopolymer stabilized bases demonstrated potential for use in flexible pavement applications at room temperature. The unconfined compressive strength of the fly ash geopolymer base material improved by 23 percent, from 13.1 MPa to 17.1 MPa. Sermsak et al. [27] reported that the 7 day unconfined compressive strength of recycled concrete aggregate, fly ash geopolymer improved as the fly ash (FA) content increased. This is because FA silica and alumina can react with calcium, resulting in pozzolan and geopolymerization products, which met the requirements for a low-traffic road. The SEM images also revealed that spherical FA and recycled concrete aggregate particles are covalently bonded, resulting in a dense matrix.

Based on the information presented above, an investigation was performed to evaluate the effects of FAG on mechanical properties as a road base stabilizer. Although several studies have been undertaken to evaluate the mechanical properties of this stabilizer, there is a lack of investigation comprising flexural strength (FS), shrinkage, unconfined compressive strength (UCS), resilient modulus (RM), and indirect tensile strength (ITS). The purpose of this study was to determine whether it would be possible and practical to make use of this material, as well as to examine the probable existence of this material in the FAG.

## 2. Materials and Methods

### 2.1. Fly Ash (FA)

**Figure 1:**
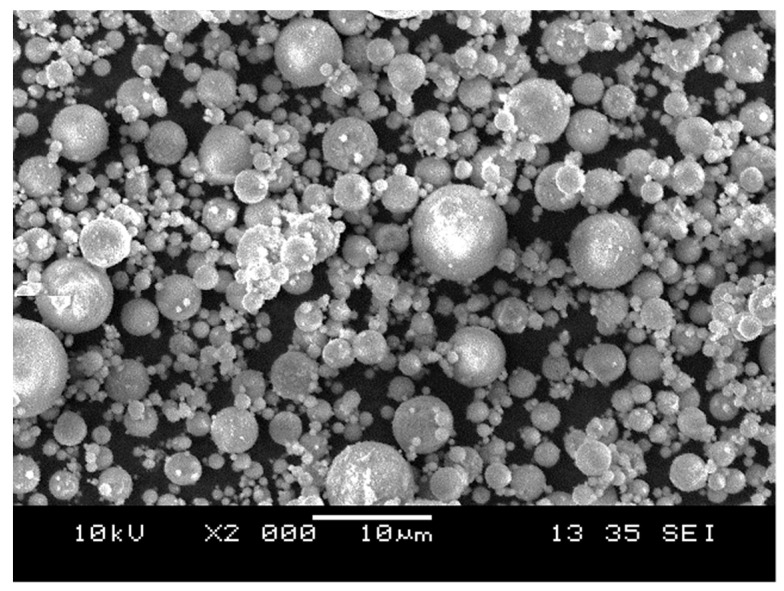
Morphology of the FA particles.

Fly ash (FA) employed in the research was acquired from Manjung Coal-Fired Power Station located in Lumut, Perak, Malaysia. The FA’s color was brown, and the fundamental chemical composition of FA was SiO_2_ (30.8%), Al_2_O_3_ (13.1%), Fe_2_O_3_ (22.99%), and CaO (22.3%), which were identified via X-ray fluorescence (XRF). The FA microstructure diagram and chemical composition have been demonstrated in Figure 1 and Table 1 accordingly.

### 2.2. Crushed Aggregate

**Figure 2:**
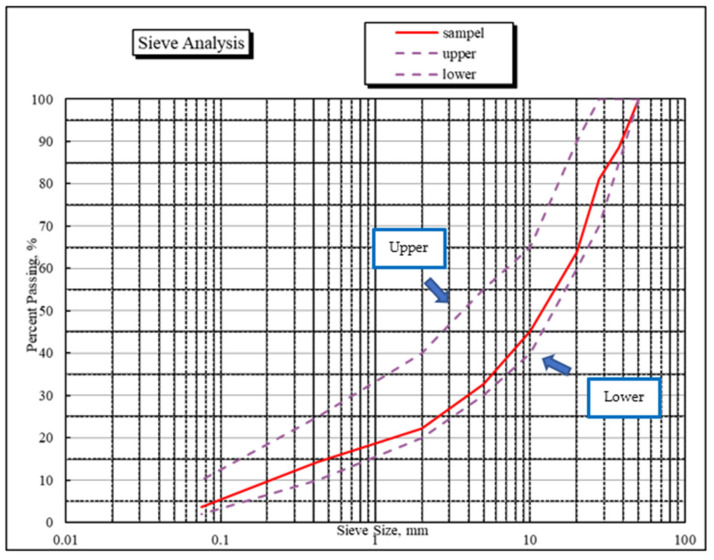
Upper and lower limit for crushed aggregate.

Crushed aggregate was obtained from Pens Industries Perlis, Malaysia. Figure 2 shows the crushed aggregate’s gradation within the upper and lower limit and meets the requirement for pavement base material by the Public Work Department (JKR), Malaysia. Note that crushed aggregate typically had nominal aggregate particles ranging roughly from quarry dust to 38 mm in size. Crushed aggregate typically had nominal aggregate particles ranging roughly from quarry dust to 38 mm in size. Los Angeles (LA) abrasion test was also conducted on a crushed aggregate sample following ASTM C131 [28]. The LA abrasion value was 25.8%. Meanwhile, the aggregate crushing value and aggregate crushing value test are 10.3% and 18.5%, respectively. The water absorption of crushed aggregate is 1.65%. These values indicated that the crushed aggregate meets the requirement for stabilized base materials of the JKR Malaysia.

### 2.3. Specimen Preparation

Prior to combining with FA, an alkaline activator, including sodium hydroxide (NaOH) and sodium silicate (Na_2_SiO_3_), was produced. In an automated mixer, the alkaline activator and FA were combined to form a homogenous paste. The technique began with a 5 min mixture of Na_2_SiO_3_ and NaOH solution. Next, FA was added to the basin and stirred for another 5 min. The Na_2_SiO_3_ to NaOH ratio was 2.5 and was fixed from previous research by Mustafa et al. [29] while the solid to liquid (S/L) ratio was 2.0. NaOH molarities of 10 M. For geopolymer sample preparation, alkali solutions with a concentration of 10 M were utilized. These concentrations were chosen based on past work and other works of literature [30].

### 2.4. Mixing Process of Fly Ash Geopolymer Concrete

The fly ash, alkali activator, and crushed aggregates were mixed until homogeneous based on the selected ratio of geopolymer paste. The FA-based geopolymer concrete was molded and compacted in a mold for several tests. The coarse and fine aggregates were taken as 80% and 85% of the whole mixture by mass. The coarse and fine aggregates utilized in this research are 45% and 55%, correspondingly, of the total aggregate. Subsequently, the remainder is the combined volume of geopolymer binder, FA, and alkaline solution. The mixed design details with different aggregate percentages of 80% (A80/G20) and 85% (A85/G15) are presented in Table 2. According to the Portland Cement Association, the amount of total aggregate used in the mixture is typically greater than 80% by mass. To obtain the maximum dry density for 95% compaction (i.e., a commonly used compaction requirement for road pavement construction), 0.5%, and 3.5% water were added to the mixtures of A80/G20 and A85/G15, respectively, for preparing the samples of unconfined compressive strength (UCS), flexural strength (FS), shrinkage, indirect tensile strength (ITS), and resilient modulus (RM).

### 2.5. Unconfined Compressive Strength (UCS) Test

The compressive strength of a mixture is assessed using the UCS test. The mixture was made using a cylindrical plastic mold that was 38 mm (diameter) × 76 mm (height) for the paste, and 101.60 mm (diameter) × 116.4 mm (height) for the FAG concrete, as per ASTM D 1633 [31]. Utilizing a hydraulic compressive strength machine, a load was continuously applied within the range of 140 to 70 kPa/s as required by ASTM D 1633 to measure the average UCS of the specimens post 7, 28, and 90 days of curing time. In addition, to simulate the exact ambient temperature in a pavement application, samples curing at room temperature were assessed in this study. To obtain the Maximum Dry Density for 95% compaction (a commonly used compaction requirement for road construction), 0.5% and 3.5% of water were added to the mixtures of A80/G20 and A85/G15, respectively, for preparing the samples of UCS and other testing in this research.

### 2.6. Flexural Strength (FS) Test

The standard test procedure ASTM C78 was used to determine the FS, which is represented as the modulus of rupture [32]. FS is measured using the usual dimensions of specimens (400 × 100 × 100) mm^3^. At a one-third distance from both beam supports, equal weights are applied. Note that FS is the maximum tensile stress attained when loading increases if a fracture occurs within the center third of the beam.

### 2.7. Shrinkage Test

The shrinkage experiment is performed at room temperature following ASTM C490 [33]. The beam specimens were chosen for the experiment, about (300 × 75 × 75) mm^3^. Correspondingly, a strain gauge is pasted on both sides of the specimen to test the straining change. Dial gauges with 0.01 mm revolutions were used to collect measurements for dimensional changes, which were then divided by the height of the specimens to obtain shrinkage or swelling values. The initial reading, known as the zero reading, was used as a standard for subsequent readings, and dial gauges were adjusted to reflect this reading. Anticlockwise readings indicate shrinking. Measurements of shrinkage values were obtained at various intervals, with more readings taken early in the curing period.

### 2.8. Indirect Tensile Strength (ITS) Test

**Figure 3:**
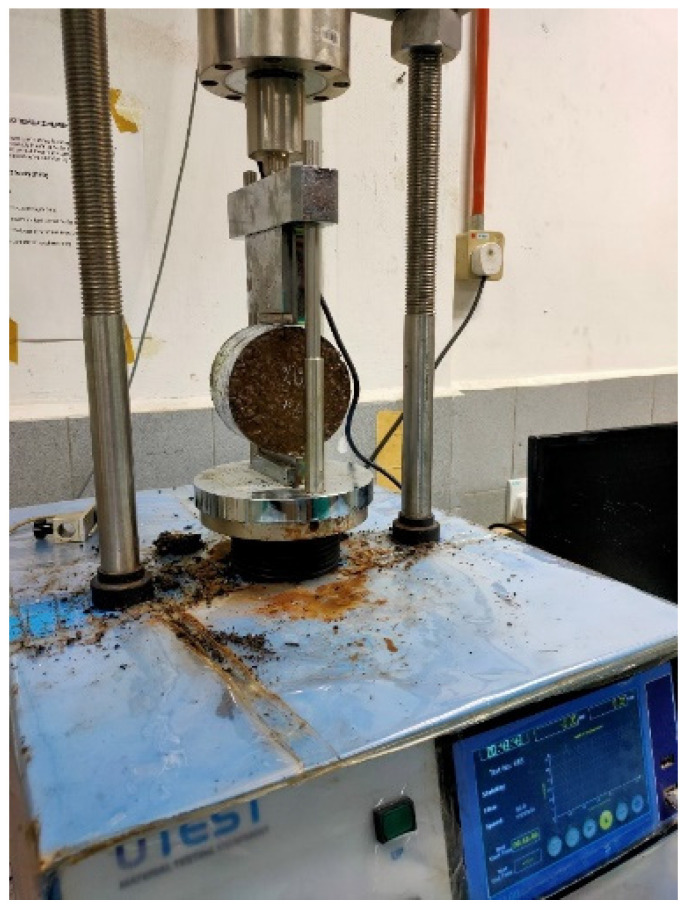
Indirect Tensile Strength Test.

The ITS test was carried out following ASTM D6931 [34]. A standard cylinder specimen with 101.6 mm (diameter) × 65 mm (height) is placed horizontally between the loading surfaces of the compression testing machine. Stabilized specimens underwent indirect tensile testing with an enforced strain rate of 1 mm/min. In this test, a compressive loading condition is applied along two axial lines to the cylindrical specimen. A constant rate of the load is used to cause failure in tension. Figure 3 shows that the load is spread out by two bearing strips to stop multiple cracks and crush at the point of load.

### 2.9. Resilient Modulus (RM) Test

**Figure 4:**
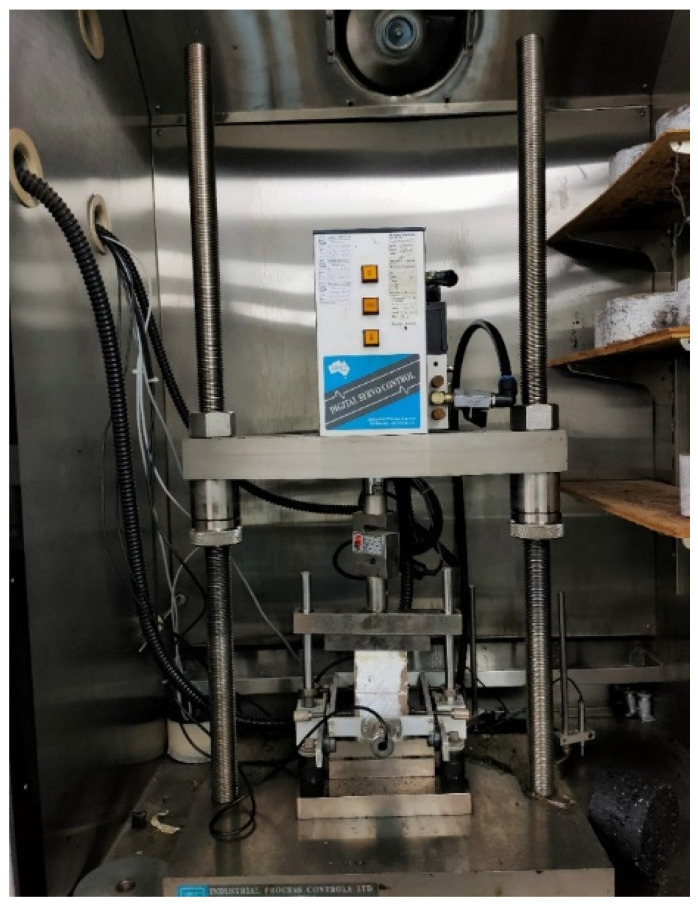
Resilient Modulus.

The RM test, as portrayed in Figure 4, has the potential to examine the materials relative quality to get a result for pavement analysis and design. Temperature, loading rate, rest intervals, and frequencies all have an impact on this test procedure. A cylindrical metal specimen that has 63.5 ± 2.5 mm height × 101.60 mm internal diameter was utilized for the mixture’s preparation. The RM of the mixtures was determined using the ASTM D 4123 [35] repeated-load indirect tension test by applying a loading frequency of 1.0 Hz and 2000 N compressive loads having a waveform at 25 °C (the appropriate load range might be 10–50% of the ITS).

## 3. Results and Discussions

### 3.1. Unconfined Compressive Strength (UCS)

**Figure 5:**
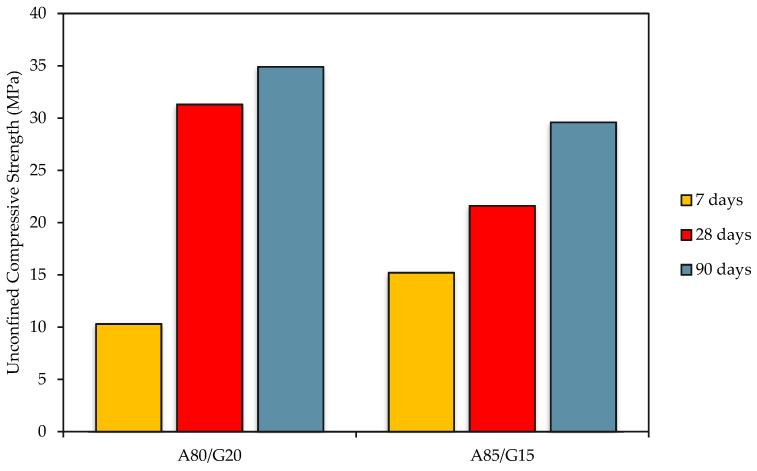
Unconfined Compressive Strength of FAG A80/G20 and A85/G15 mixtures at 7, 28, and 90 days.

The traffic loads transferred from the asphalt surface course are sustained by the base course layer, which serves as the primary load-bearing course in pavement constructions. Therefore, strength is the primary characteristic of the base layer. It is commonly acknowledged that the unconfined compressive strength (UCS) is a crucial signal for determining the tensile and shear characteristics of the base layer. The UCS of FAG concrete is measured at aging days of 7, 28, and 90, as presented in Figure 5. The graph presents FAG concrete with different total aggregate content, named A80/G20 and A85/G15. UCS values for 7, 28, and 90 days cured specimens were determined to be within 10.3 to 34.9 MPa for both mixtures.

The lowest UCS (10.3 MPa) was for A80/G20 at 7 days, which has 80% crushed aggregate at the geopolymer content of 20%. In contrast, the mixture with the same content has the highest value of UCS (34.9 MPa) at 90 days. A mixture of A85/G15 has a range of strength values of 15.2 MPa at 7 days to 29.6 MPa at 90 days. The minimum requirement to be fulfilled is 5 MPa for 7 days and 10 MPa for 28 days, according to Public Work Department (JKR), Malaysia for a stabilized base [36]. The results indicated that the strength value at A85/G15 was lower than A80/G20 at 28 days and 90 days.

It is believed the result had related to the presence of additional water of 3.5% from the compaction test to the mixtures of A85/G15 and had caused a reduction in the availability of geopolymer matrix. From this figure, it is evident that adding water content significantly reduces the strength. This result was supported by Aliabdo et al. [37]. The mixtures containing 15% (A85/G15) alkaline activator with the addition of water exhibited comparatively less strength than those that contained 20% (A80/G20) of the alkaline activator with the addition of water. When more water was added to the A85/G15 mixes, it raised the water-to-solid ratio (*w*/*s*) and reduced the alkaline activator solution’s concentration, resulting in a drop in strength.

The UCS increased at both mixtures due to increased chemical reactions during the geopolymerization process. FA contains high silica (Si) and alumina (Al) content, as discussed in the chemical composition analysis. In geopolymerization, FA serves as a source of reactive Si and Al to form silicate and aluminate hydrates, which are important for developing strength. The high presence of Si and Al in FA reacted with calcium (Ca) and generated calcium aluminate silicate hydrate (C-A-S-H) eventually improve its mechanical properties.

The improvement in UCS with crushed aggregate in the mixture is due to improved load transmission in the road base between the mixture’s particles. More precisely, greater aggregate percentages generate higher degrees of friction and interlocking processes between aggregate particles and the geopolymer binder. As a result, the pavement’s capacity to withstand strong traffic loads has been much improved, both in the magnitude and frequency of wheel loads.

The UCS of FAG concrete is around 50% greater than that reported by Dong et al. [38] who indicated that the UCS of cement-stabilized base materials varied from 5 to 7 MPa after 7 days.

### 3.2. Flexural Strength (FS)

**Figure 6:**
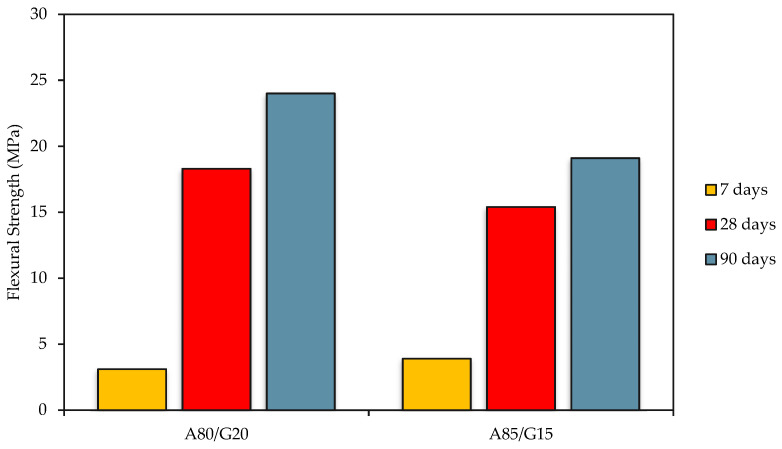
Flexural Strength for FAG A80/G20 and A85/G15 mixtures at 7, 28, and 90 days.

The capacity of a material to withstand deformation under applied stress is measured by its flexural strength (FS) (or rupture modulus). The test findings of FS are presented in Figure 6. Other than that, the FS of the mixtures changed substantially under three different curing times of 7, 28, and 90 days, as indicated by the data displayed. It has been discovered that the pattern of variation in UCS and the pattern of variation in FS are largely consistent. The FS of FAG concrete base materials constantly rises as curing times increase, although at varying rates.

For different curing days, the strength of A80/G20 mixes ranges from 3.1 MPa to 24.0 MPa, whereas the strength of A85/G15 mixtures ranges from 3.9 MPa to 19.1 MPa. FS boosted promptly, specifically in the 7 to 28 day curing periods, while it most likely increased slowly throughout the curing periods of 28 to 90 days. In the standard procedure of stabilized base from the JKR Malaysia, the FS requirement is 2.0 MPa at 28 days [36].

The FS value of A85/G15 mixtures was lower than A80/G20 mixtures due to an increase in aggregate content. The possible reason is the weak interface bond strength between geopolymer binders and crushed aggregate for A85/G15 compared to A80/G20 mixtures, as seen in Figure 6. From the presented graph, the age effect of FAG concrete on the FS is due reactivity of the aluminosilicate precursors of the geopolymer gel, which is resulted from the substantially fast geopolymerization process.

A mixture of A85/G15 at 28 and 90 days is related to the presence of 3.5% additional water and has caused the reduction of the availability of the geopolymer matrix. The strength gradually diminished as a result of its increased water-to-solids ratio (*w*/*s*) and decreased alkaline activator solution concentration. Even with just 0.5% of water and an alkaline activator, the strength increased significantly. A comparatively less strength was displayed by the mixtures comprising 15% alkaline activator and 3.5% water compared to those with 20% alkaline activator and 0.5% water.

Furthermore, the rapid increase in flexural strength of FAG was approximately 83% after curing between 7 and 14 days. Additionally, it was demonstrated that aging time has a significant impact on how well a geopolymer develops its strength. This is caused by the FA’s high iron (Fe_2_O_3_) concentration, which is about 22.99%. In the geopolymer system, the reaction between Fe_2_O_3_ FA and the alkali solution takes some time before the iron silicate binder gel is produced. Compared to Si and Al, iron has a large atomic diameter and a high atomic mass. Therefore, the geopolymer’s resilience is aided by ferrosialate in addition to the sodium aluminosilicate gel. As a result of the prolonged curing time, the strength of concrete supplied by iron oxide has increased [39].

The treated layers in the pavement system are subject to flexural stress when a load is applied. Hence, the FS of FAG concrete mixtures is a crucial quality. The treated layers must be considered for fatigue cracking, much like the repetitive load application in the pavement system. The assessment of fatigue life for such mixtures is heavily influenced by flexural stiffness. Note that FS is an essential performance parameter for assessing the crack resistance of a cementitious-stabilized layer subjected to loading.

### 3.3. Shrinkage Analysis

**Figure 7:**
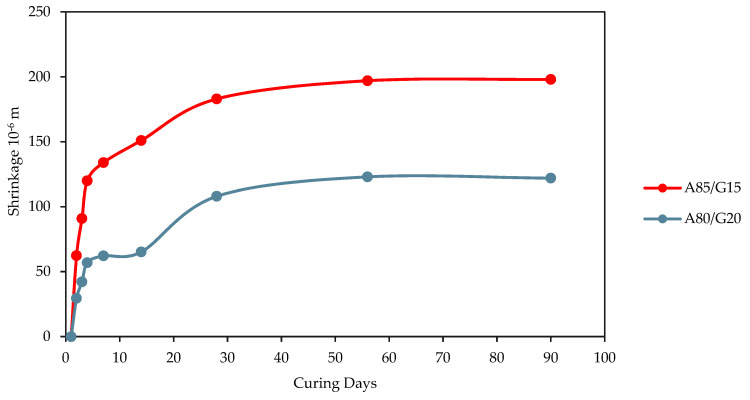
Shrinkage value for A80/G20 and A85/G15 mixtures.

Shrinkage increases rapidly during the first 7 days, and the increase rate is slower in the subsequent days. From Figure 7, it is reasonable to conclude that the drying shrinkage shows an increment in the drying period for the type of blend mixtures that were tested, ranging from 0 to 90 days.

The evaluation reveals that the presence of crushed aggregate material, and consequently, geopolymer binder, has a substantial impact on the potential for shrinkage. For example, the mixture blend that was prepared with 80% crushed aggregate material and treated with 20% geopolymer content (A80/G20) showed a lower drying shrinkage value, which is up to 122 × 10^−6^ m at 90 days of curing compared to the mixture A85/G15 value of 198 × 10^−6^ m at the same number of curing days. This is because the A80/G20 mixture was treated with 20% geopolymer content. However, beginning on day 56, the shrinkage value remained constant for both mixes.

The available water consumption in the hydration reaction of the mixture is the primary cause of the increment in drying shrinkage that occurs in conjunction with the presence of geopolymer content. Therefore, an increase in the aggregate content of the materials for stability may result in a high moisture consumption and, as a direct consequence of this, a significant degree of shrinkage, followed by cracking of the material. For example, the mixture of A85/G15 had a high-water absorption percentage compared to A80/G20 because the crushed aggregate content of A85/G15 was greater than A80/G20; thus, there is an excess of moisture during evaporation of A85/G15 that leads to high shrinkage compared to A80/G20 mixtures. The other reason of the lowest shrinkage is the water added to the mixture of A80/G20 was lower than mixture A85/G15 which is 0.5% and 3.5% respectively.

The moisture content is an important component in reducing the degree of shrinkage of stabilized materials since it is mostly induced by moisture loss due to hydration or evaporation. Increasing the moisture content causes more moisture to evaporate, hence a greater magnitude of shrinkage stresses. This was related to an excess of moisture that was not required for hydration when the higher moisture content was used. Therefore, it is required to control the moisture content and preferably to use the Optimum Moisture Content in the compaction test. When compared to the traditional base materials, the FAG base material’s dry shrinkage value was found to be slightly lesser, suggesting that the usage of geopolymer as a stabilizing agent potentially helps alleviate reflective cracks.

### 3.4. Indirect Tensile Strength (ITS)

**Figure 8:**
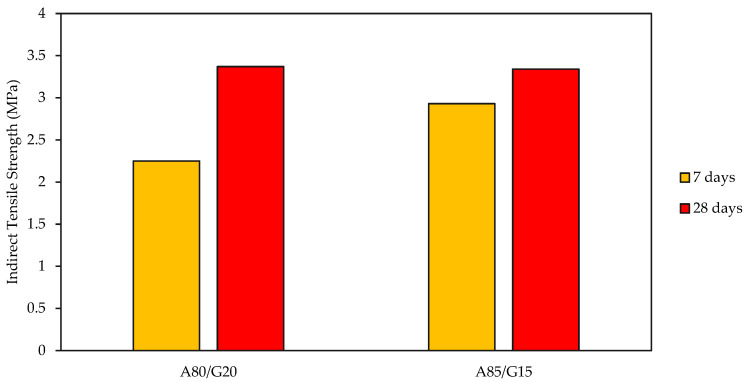
Indirect Tensile Strength for A80/G20 and A85/G15.

Under traffic loads, the base course of pavement constructions is subjected to tensile stresses and strains. Figure 8 illustrates the indirect tensile strength (ITS) test findings for the specimens prepared in the range of A80/G20 and A85/G15 (crushed aggregate and geopolymer content) and cured for the period of 7 and 28 days before testing. For mixture A80/G20, the increasing percentage is about 49.7% for curing 7 to 28 days. Meanwhile, about a 14% increase percentage was observed for mixture A85/G15 in 7 to 28 days of curing.

The development rate of indirect tensile strength, in general, is proportional to the amount of geopolymer used. This is because high Ca in FA content in the mixture can contribute to the strength and binding effect. With increasing volumes of crushed aggregate and curing time, the value of ITS increased consistently. Like the UCS, the reduction in ITS is more significant at greater geopolymer levels than at low ones. In general, the ITS values for the different examined specimens remained within a range of 12 to 16% of the UCS values. The addition of FA enhanced the indirect tensile strength, and so did the decrease of porosity. Reducing the porosity increases the contact between the particles, boosting their bonding strength.

### 3.5. Resilient Modulus (RM)

**Figure 9:**
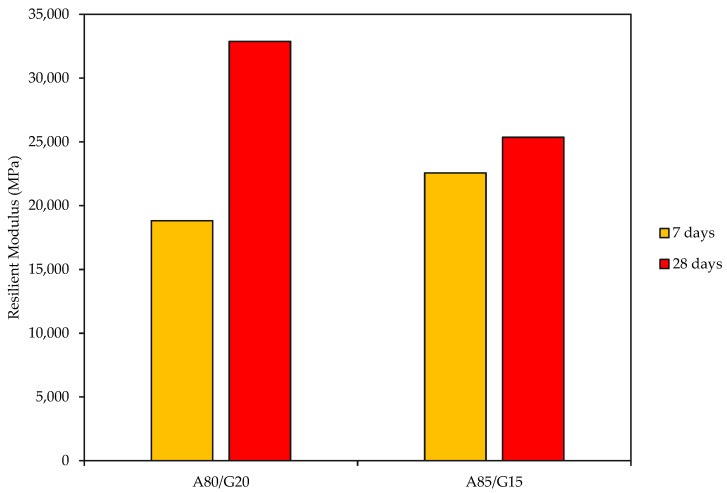
Two Resilient Modulus for A80/G20 and A85/G15 mixtures.

The important mechanical parameter utilized to examine the pavement materials’ performance is its resilient modulus (RM), which is essential for calculating stresses, strains, and displacements within pavement structure layers susceptible to traffic-induced loads. The resilient modulus test outcomes are shown in Figure 9 for mixtures cured for 7 and 28 days. The figure shows that the A80/G20 combination had the greatest RM (32,881 MPa) and the lowest RM (18,819.3 MPa) at 28 days and 7 days, respectively, with A85/G15 mixtures in between. This strong connection between these two mixtures is consistent with the results of flexural strength.

Strong support for the upper asphalt courses and even distribution of vehicle wheel loads in the lower subgrade are provided by the high stiffness stabilized base layer. While materials having high stiffness have a tendency to become brittle, which raises the tensile condition of the layers and leads to early failure, excessive base stiffness may have a detrimental influence on the service life of the pavement. Therefore, the effect of the base layer modulus on the performance of the pavement has been studied by numerous academics [40]. The findings showed that boosting the base layer’s modulus could significantly reduce tensile stress at the base of asphalt courses and pavement deflection, resulting in a more significant economic benefit by lowering the overall thickness of pavement structures.

## 4. Conclusions

In this research, mechanical parameters were used to evaluate how well FAG performed as a road base, for instance, flexural strength (FS), indirect tensile strength (ITS), resilient modulus (RM), shrinkage, and unconfined compressive strength (UCS) with different percentages of crushed aggregates. The mixture of A80/G20 and A85/G15 was used to determine the performance evaluation of the pavement base. The following conclusion can be summarized.

- The major finding is that only 80% and 85% of crushed aggregates are appropriate for usage as a road base material as per the compaction test result. The increase in strength value is due to the friction and interlocking actions between the aggregate particles and the geopolymer binder, which can enhance the load transfer in the road base layer. From the results obtained, the UCS values for 7, 28, and 90-days cured specimens were in the range of 10.3–34.9 MPa for all mixtures.
- The flexural strength of FAG concrete base grew continuously as curing times increased, and the strength findings followed the same pattern.
- FAG base material was discovered to have a slightly less dry shrinkage value than conventional base materials, suggesting that using geopolymer as a stabilizing agent may assist in improving reflective cracks. Additionally, it is one of the elements influencing how well the pavement performs.
- For the results of the indirect tensile strength of the A80/G20 mixture, the percentage increase was about 49.7% for the 7 to 28 day curing period. The growth percentage of the A85/G15 mixture increased by 14% during the 7 to 28-day curing period.
- The A80/G20 mixture obtained the highest resilient modulus of 32,881 MPa, and the same mixture also recorded the lowest is 18,819.3 MPa. These two mixtures magnitudinal relationship reflects the findings of the UCS test.

The above conclusions have verified that incorporating FAG as a road base stabilizer is conducive to promoting environmental sustainability. However, further research is essential to examine the properties and performance of geopolymer road bases using the geopolymerization method using different raw materials.

## Figures and Tables

**Table 1:** The FA chemical composition (%).

Composition	Weight (%)
SiO_2_	30.80
CaO	22.30
Fe_2_O_3_	22.99
Al_2_O_3_	13.10
MgO	4.00
K2O	1.60
TiO_2_	0.89
SO_3_	2.67
MnO	0.21
LOI	1.44

**Table 2:** Mix proportion of FAG concrete having different aggregate percentages.

Sample Name	NaOH Concentration (M)	S/L Ratio	Na_2_SiO_3_/NaOH Ratio	Percentage of Aggregate (A)	Percentage of FAG (G)
A80/G20	10 M	2.0	2.5	80	20
A85/G15			2.5	85	15

## Data Availability

Not applicable.
